# Non-Coding RNAs as Biomarkers of Tumor Progression and Metastatic Spread in Epithelial Ovarian Cancer

**DOI:** 10.3390/cancers13081839

**Published:** 2021-04-12

**Authors:** Karolina Seborova, Radka Vaclavikova, Lukas Rob, Pavel Soucek, Pavel Vodicka

**Affiliations:** 1 Toxicogenomics Unit, National Institute of Public Health, 100 42 Prague, Czech Republic; karolina.seborova@szu.cz (K.S.); radka.vaclavikova@szu.cz (R.V.); pavel.soucek@szu.cz (P.S.); 2 Biomedical Center, Faculty of Medicine in Pilsen, Charles University, 323 00 Pilsen, Czech Republic; 3 Department of Gynecology and Obstetrics, Third Faculty of Medicine and Faculty Hospital Kralovske Vinohrady, Charles University, 100 42 Prague, Czech Republic; lukas.rob@fnkv.cz; 4 Institute of Experimental Medicine, Czech Academy of Sciences (CAS), 142 20 Prague, Czech Republic; 5 Institute of Biology and Medical Genetics, First Faculty of Medicine, Charles University, 120 00 Prague, Czech Republic

**Keywords:** ovarian cancer, progression, metastasis, ncRNA, miRNA, lncRNA, biomarkers, epigenetics

## Abstract

**Simple Summary:**

Despite advances in cancer research in recent years, efficient predictive biomarkers of tumor progression and metastatic spread for ovarian cancer are still missing. Therefore, we critically address recent findings in the field of non-coding RNAs (microRNAs and long non-coding RNAs) and DNA methylation in ovarian cancer patients as promising novel biomarkers of ovarian cancer progression.

**Abstract:**

Ovarian cancer is one of the most common causes of death among gynecological malignancies. Molecular changes occurring in the primary tumor lead to metastatic spread into the peritoneum and the formation of distant metastases. Identification of these changes helps to reveal the nature of metastases development and decipher early biomarkers of prognosis and disease progression. Comparing differences in gene expression profiles between primary tumors and metastases, together with disclosing their epigenetic regulation, provides interesting associations with progression and metastasizing. Regulatory elements from the non-coding RNA families such as microRNAs and long non-coding RNAs seem to participate in these processes and represent potential molecular biomarkers of patient prognosis. Progress in therapy individualization and its proper targeting also rely upon a better understanding of interactions among the above-listed factors. This review aims to summarize currently available findings of microRNAs and long non-coding RNAs linked with tumor progression and metastatic process in ovarian cancer. These biomolecules provide promising tools for monitoring the patient’s response to treatment, and further they serve as potential therapeutic targets of this deadly disease.

## 1. Introduction

Ovarian cancer (OC) is a complex heterogeneous malignant disease and the second cause of female cancer deaths attributed to gynecological tumors. According to GLOBOCAN 2018, OC is the eighth most common cancer (both in incidence and mortality) worldwide among women [1]. Tumors of epithelial origin represent the most frequently diagnosed OC type (90%) and comprise type I (endometrioid, mucinous, clear cell, and low-grade serous ovarian carcinomas) and type II that includes carcinosarcomas, undifferentiated carcinomas, and high-grade serous ovarian carcinomas (HGSCs), accounting for 70% of OC cases [2,3]. The disease is usually diagnosed at an advanced stage (FIGO III and IV) when the 5-year survival rate is approximately 20–45%, while it is 40–70% for stage I. While localized disease exhibits a 5-year survival rate of 80%, the advanced disease has a 5-year survival rate of only 30% [4,5].

Most of the OC patients undergo chemotherapy combining platinum derivatives (carboplatin and cisplatin) and taxanes (paclitaxel and docetaxel) [6]. New therapeutic approaches have been introduced to the therapy of OC recently, e.g., poly(ADP-ribose) polymerase inhibitors (PARPi), such as olaparib, or antiangiogenic agents such as bevacizumab or pazopanib [7,8].

Nowadays, among the main diagnostic tools, we count physical examination, serum level of oncomarker CA125 (or HE4 in some cases), computed tomography, and transvaginal ultrasonography [9,10]. The lack of reliable biomarkers for early OC detection enables diagnosis only due to non-specific symptoms when the disease is already in progress. Late diagnosis at advanced stages of OC is related to chemotherapy resistance and the development of metastases. Progression of OC and metastatic spread involves the dissemination of tumor cells from primary tumors.

Molecular characteristics of OC subtypes, based on genetic variability, have been described a few years ago. Rojas et al. summarized specific gene mutation profiles for Type I OC (mutations in the MAPK pathway—*KRAS*, *BRAF*, *PTEN*, *CTNNB1*, etc.) and Type II OC (*TP53*, *BRCA1*, *BRCA2*, *KIT*, and *EGFR*) that could represent novel therapeutic targets or biomarkers of therapeutic efficacy [3]. Clinicopathological and molecular characteristics of Type I and Type II OC subtypes correspond with prognosis and response to treatment [11]. As recently overviewed, DNA repair machinery also plays an important role in the OC development risk, prognosis, and therapeutic outcome [12].

**Figure 1:**
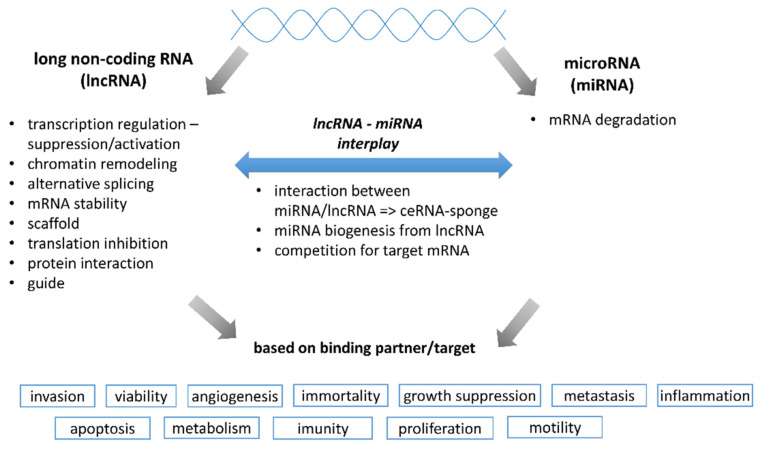
Simplified overview of lncRNA and miRNA functional properties. This figure shows possible interacting molecular determinants for lncRNA and miRNA as well as a wide range of cellular processes influenced by lncRNA/miRNA interactions with target binding partners.

Although the importance of genetic variation and mRNA expression profiles was already well characterized [3,10], the roles of regulatory elements as non-coding RNAs (ncRNAs) and epigenetic regulation (including DNA methylation and histone or chromatin post-translational modifications) became recently broadly studied thanks to the advent of new experimental techniques. Over 98% of the human transcriptome lacks protein-coding capacity. Despite this fact, the non-protein-coding transcriptome has an important role in cellular processes, including chromatin remodeling, transcription, post-transcriptional modifications, and signal transduction [13]. Non-coding RNAs, referred to as ncRNAs, are a part of networks influencing cell proliferation, invasion, migration, EMT, angiogenesis, DNA repair, cell signaling, and, finally, diverse physiological programs with developmental and oncological contexts. ncRNAs (miRNAs, circular RNAs-circRNAs, siRNAs, piRNAs, and lncRNAs) regulate the expression of protein-coding genes or interact with other species of ncRNA and regulate their stability. ncRNA may interact with other non-coding elements through mechanisms, including sequestration. Figure 1 represents a simplified scheme of general ncRNAs functional properties.

In this review, we focus our attention on regulatory elements belonging to ncRNAs (particularly microRNA (miRNAs) and long non-coding RNAs (lncRNAs)) and their associations with the progression and development of OC metastases investigated using both in vitro models and patient tumor tissue specimens.

## 2. Progression of Ovarian Carcinoma and Development of Metastases

**Figure 2:**
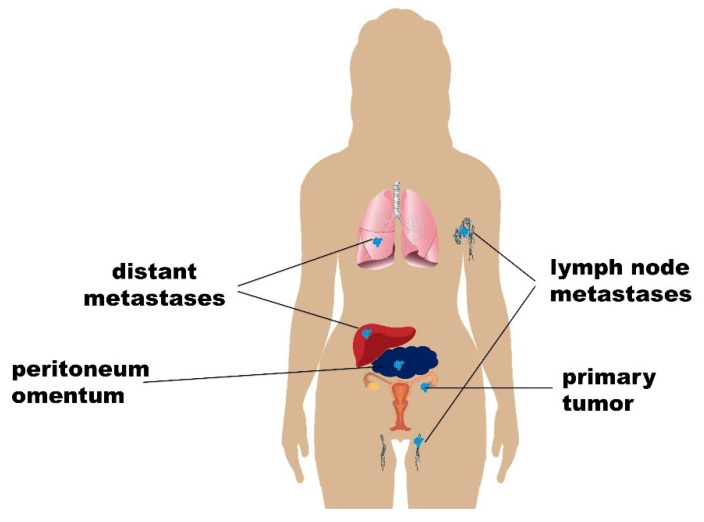
Metastatic routes of ovarian cancer (OC) metastasis. This scheme shows different metastatic scenarios in OC. The transcoelomic/peritoneal route is the main route in OC metastases development, especially into the peritoneum/omentum. During the subsequent progression, metastases in distant lymph nodes and organs (lungs or liver) are developed.

The metastatic process consists of the following steps: (i) separation of tumor cells from the primary tumor site; (ii) escape of tumor cells to the circulatory system (blood, lymph); (iii) adhesion of tumor cells to the endothelium of a vessel wall, followed by their evasion from the circulatory system and formation of the new tumor loci; and (iv) proliferation and tumor growth of metastatic cells in a new location. OC metastases disseminate throughout the body via peritoneal, hematogenous, and lymphatic routes. Most metastatic lesions affect the peritoneum/omentum or abdominal organs by the most prevalent peritoneal route. Tumor cells also often infiltrate pelvic lymphatic nodes with subsequent metastatic spread to the liver or lungs (Figure 2). Essential steps of the metastatic process have been in detail reviewed in several reviews, see, for example, in [14,15,16]. In a study by Deng et al. on 1481 OC patients, the liver was identified as the most common distant site of metastases development, followed by lymph nodes, lungs, bones, and brain [17]. Molecular determinants of individual steps in the metastatic development and spread are linked with the following genes. First step—metastasis initiation of transcoelomic route is defined by loss of cell adhesion, characterized by key genes (*CDH1*, *CDH2*, and *UPAR*) [18,19], epithelial–mesenchymal transition (EMT) (*ZEB1*, *ZEB2*, *SNAI1*, and *SNAI2*) [20], migration (*UPA*) [21], and spheroid formation (*HGF*) [22]. The next step is metastasis progression characterized by dissemination in the peritoneal cavity (*VEGFA*, *MMP2*, *MMP9*, *CXCRL4*, *CXCL12*, and *CD44*) [23,24], resistance to anoikis (*RAB25*) [25], and evading apoptosis/immunity. The last step—metastasis spread, starts with attachment to the peritoneal surface (*MMP2*, *MMP9*, *MMP14*, *CDH1*, and *LPA*) [26,27,28], formation of metastases (*UPAR* and *SPINK13*) [29], and subsequent invasion (*DNMT1*, *MAPK1*, *MAPK3*, and *ETS1*) [30,31], and angiogenesis (*VEGFA*, *IL1*, and *IL6*) [32,33]. The hematogenous route of metastases development is more prevalent than formerly estimated [34].

Adherent epithelial cells are transformed into invasive mesenchymal cells during EMT, which leads to the separation and spread of tumor cells from primary tumors. In general, three types of EMT are recognized: type I—embryogenic EMT, type II—EMT associated with inflammation and tissue damage, and type III—EMT involved in tumor progression [35]. Deregulation of E-cadherin, N-cadherin, and vimentin expression are characteristic EMT markers. Vimentin, encoded by the *VIM* gene, is a filament protein with structural functions and vital importance for cell migration and proliferation [36,37], and cadherins are transmembrane proteins that mediate cell–cell adhesion and play a crucial role in tissue morphogenesis and homeostasis [38]. Expressions of the above EMT markers are regulated by transcription factors from ZEB (*ZEB1* and *ZEB2*), SNAIL (*SNAI1* and *SNAI2*), and TWIST families [39,40,41,42]. Expression of these factors, and other EMT markers, is regulated by epigenetic modifications (DNA methylation and histone modifications) and by non-coding transcript elements [43,44,45,46,47,48].

The tumor microenvironment strongly impacts the metastatic process. It concerns particularly cancer-associated fibroblasts (CAFs), which produce several growth factors, e.g., EGF (epidermal growth factor) and HFG (hepatocyte growth factor) that promote tumor growth and progression [22,49]. Vascular endothelial growth factor (VEGF) is a cytokine essential for angiogenesis and lymphangiogenesis. The interaction between tumor and stromal cells can result in an increased VEGF expression [50,51]. Matrix metalloproteinases (MMPs) [52] and hypoxia-inducing factor-1 (HIF-1), inducing EMT in a hypoxic tumor environment, represent additional important factors in metastasis [53,54,55]. In the current chapter, we briefly described individual steps of EMT and thus metastases development. Many other molecules/signaling pathways are involved in this complex and crucial process.

An increasing number of molecular factors are is important for tumor progression, and the metastatic process is currently subject to discovery and further validation. Among these factors, regulatory non-coding elements, as microRNAs (miRNAs) and long non-coding RNAs (lncRNAs), seem to arise as potential biomarkers of metastases development.

## 3. The Role of Non-Protein-Coding Transcripts and Their Role in the Development of Ovarian Cancer Metastasis

**Figure 3:**
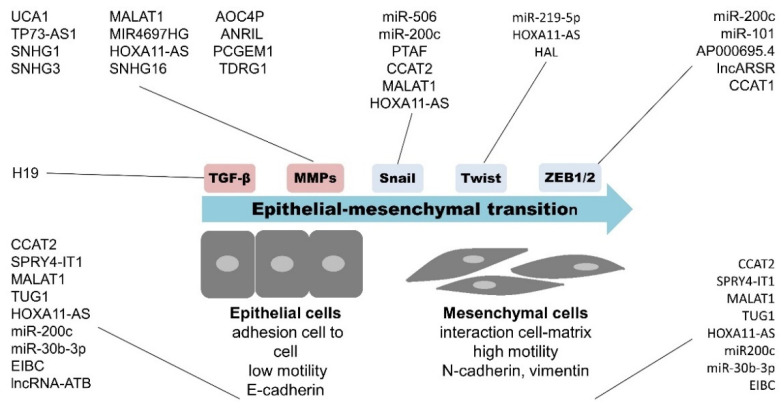
Schematic representation of ncRNA influencing epithelial–mesenchymal transition (EMT) process in OC. This chart shows ncRNAs elements—miRNAs and lncRNAs, which associate with EMT regulation. During EMT, adherent epithelial cells are changed to highly motile mesenchymal cells, capable of invading other places in human body. Few key molecules, i.e., E-cadherin, N-cadherin, and vimentin, whose expression is regulated by miRNAs and lncRNAs, characterize this process. Several ncRNAs regulate/interact with EMT-transcription factors (Snail, Twist, and ZEB families) and thus affect the EMT process. Other key players in EMT regulated by ncRNA are metalloproteinases (MMPs) and TGF-β.

ncRNAs play substantial roles in the regulation of gene expression at both transcriptional and post-transcriptional levels. Due to this large functional importance, ncRNAs represent a promising potential as clinically relevant cancer biomarkers and therapeutic targets in OC [13,56,57,58,59]. The role of ncRNAs in the regulation of the EMT process seems especially crucial in the context of OC progression and the development of metastases. miRNAs, particularly the miR-200 family—miR-101 [44,60], miR-219-5p [61], and miR-506 [62]—were demonstrated as effective regulators of EMT in OC through targeting and subsequent regulation of EMT-transcription factors (SNAIL, TWIST, and ZEB families). The roles of other non-coding entities such as, e.g., lncRNAs, are not yet fully understood due to publication of the first data in the last few years. lncARSR and CCAT1 seem to regulate the expression of the ZEB family [63,64]; HOXA11-AS and CCAT2 rule the TWIST family [45,46]; and PTAF, CCAT2, and MALAT1 the SNAIL family [65]. The schematic representation of ncRNAs implicated in the OC EMT process is in Figure 3. Taken together, out of all the ncRNA elements recognized to date that are important for the whole process of OC progression and metastases development, miRNAs and lncRNAs are the most attractive.

### 3.1. microRNA (miRNA) in Ovarian Cancer Progression

miRNAs are 19–25-nucleotide-long RNAs interacting with target mRNA based on sequence complementarity between them. The binding of miRNAs to their target gene sequences in the 3′-untranslated region (3′UTR) leads to the mRNA degradation or repression of mRNA translation (reviewed in [66]). miRNAs are functionally involved in proliferation pathways, apoptosis machinery, EMT process, or angiogenesis, and their expression is often deregulated in a variety of diseases, including cancer [66,67,68,69]. In cancer, miRNAs can act as tumor suppressors (e.g., miR-1299 and miR-506) or oncogenes (e.g., miR-125b), based on their binding partners [62,68,70].

More specifically, the well-known miRNA-200 family consisting of miR-200a, miR-200b, miR-200c, miR-141, and miR-429 regulates the EMT process, critical for OC progression via governing the expression of EMT transcription factors such as *ZEB1*, *ZEB2*, and EMT markers; E-cadherin; and vimentin in ovarian cancer cells [44,71,72]. Upregulation of miR-200a led to the silencing of important tumor suppressor gene *PTEN* in the OVCAR3 cell line in vitro [73]. Additionally, miR-506, interacting with transcription factors *SNAI1* (indirectly) and *SNAI2* (directly) or vimentin and N-cadherin, led to the inhibition of the EMT process. On the other hand, the interaction of miR-506 with TGF-β, another regulator of the EMT process, resulted in a TGF-β-induced EMT [62,74]. Another miRNA with a possible role in EMT regulation, miR-122, was related to expression changes of major EMT markers, i.e., E-cadherin, vimentin, and metalloproteinases (*MMP2* and *MMP14*) [75].

Only a few studies comparing global miRNA expression profiles (miRNOM) of primary OC tumors and metastatic tissues are available. The expression profiles of overall miRNOM using microarray technologies revealed cassette of upregulated (miR-210, miR-182, miR-200c, miR-23a, and let-7f) and downregulated (miR-145 and miR-214) miRNAs in OC effusions in comparison to OC primary tumors [71]. Another study, comparing nine pairs of primary serous OC tumors and omental metastases, reported seventeen differentially expressed miRNAs. Particularly miR-21, miR-150, and miR-146a were upregulated in omental metastases, suggesting the role in their metastases and chemotherapy resistance [76]. Using RNA-seq technology, Bachmayer-Heyda et al. [77] identified a profile of other 11 miRNAs associated with the metastatic spread of the HGSC OC tumor subtype (miR-706, miR-804, miR-1003, miR-1254, miR-1628, miR-1927, miR-2353, miR-2503, miR-2916, miR-3475, and miR-3784). Nevertheless, most studies focused on individual miRNAs, as summarized in Table 1. Concerning OC blood samples, miRNAs belong to the most studied entities in plasma or serum samples of patients. A higher level of miR-590-3p in OC plasma samples compared to control ones was observed [78], and lower expression of miR-145 in serum samples from OC patients (*n* = 70) was associated with disease progression [79]. Zuberi et al. observed an association between high expression of miR-125b in serum and the presence of OC lymph node and distant metastases [80]. Thus, based on the recently deciphered role of miRNAs in OC progression, future research in this direction is highly encouraged.

The role of miRNAs is further modulated by genetic variability, e.g., single nucleotide polymorphisms (SNPs) in miRNAs or target genes, and especially, changes in miRNA binding sites, which may have a pathological impact. SNP in the 3′UTR region of the *MDM4* gene (rs4245736, A > C) results in the creation of a new binding site for miR-191, and the presence of a C allele reduces the risk of OC development and suppresses its progression [81]. Another SNP rs11614913 (CC genotype) in miR-196a-2 increases the risk of OC and promotes cell migration and invasion [82]. Nevertheless, in a large study conducted on 3973 patients with invasive OC and 3276 control subjects, the authors have selected previously identified SNPs with OC risk association. The authors studied 226 SNPs localized within 15 kb of miRNAs processing genes *DDX20*, *DROSHA*, *GEMIN4*, or *XPO5* and 23 SNPs in binding sites of miRNA targets (*CAV1*, *COL18A1*, *E2F2*, *IL1R1*, *KRAS*, and *UGT2A3*). No association between these SNPs and OC risk was observed after adjustment for European ancestry. This study shows the need for result validation on large study cohorts, not only in genetic variability studies [83]. As shown above, genetic variability in sequences crucial for the function of particular miRNAs should also be considered in the study of OC development and progression.

In recent years, functional in vitro and in vivo studies suggested a function of miRNAs in OC progression. The investigators observed the effects of particular miRNAs on the proliferation, migration, and invasive potential of tumor cells. For example, overexpression of miR-542-3p, miR-219-5p, miR-532, and miR-3064 suppressed the proliferation of OC cell line models in vitro [61,84,85,86]. The expression of miRNAs seems to be associated with cell migration. For example, overexpression of miR-222 and miR-205 enhances cell migration [87,88]. miRNAs can modulate the invasive behavior of OC cells, e.g., miR-106 inhibition suppresses cells invasion, miR-376a overexpression stimulates cell invasion, and, on the other hand, miR-145 overexpression suppresses cell invasion [89,90,91]. More miRNAs with an implicated role in OC are summarized in Table 1, including observed deregulation and predicted targets. More detailed information on miRNAs and their possible functions and roles in cellular processes, such as cancer cell proliferation, migration, and invasion, including experimental models and interactions with other molecules and target genes, is summarized in Table S1.

**Table 1:** miRNAs and their implication in ovarian cancer progression.

	Experimental Model				
miRNA	Patient Samples	Cell Lines, Xenografts	Deregulation	Target	Reference
miR-9	✓	✓	↓	NF-κB *	[92]
miR-23b	✓	✓	↓	RUNX2 *	[93]
miR-30b-3p	-	✓	↓	E-cadherin, β-catenin, vimentin **, CTHRC1 *	[43]
miR-101	-	✓	-	ZEB1, ZEB2 *	[60]
miR-106a	✓	✓	↑	PTEN **	[90]
miR-106b	✓	✓	↓	RhoC **	[94]
miR-122	-	✓	-	P4HA1 *	[75]
miR-138	✓	✓	↓	SOX4, HIF-1α *	[95]
miR-141	✓	✓	↑	KLF12 *	[96]
miR-145	✓	✓	↓	MTDH *	[91]
miR-193b	✓	✓	↓	uPA *	[30]
miR-199a-5p	-	✓	↓	NF-κB1 *	[97]
miR-200a	✓	✓	↑	PTEN *	[73]
miR-200c	-	✓	↓	ZEB1, vimentin, E-cadherin **	[44]
	-	✓	-	HOTAIR, Snail, E-cadherin **	[98]
miR-204	✓	✓	-	BDNF *	[99]
miR-205	✓	✓	↑	SMAD4, PTEN *	[88]
miR-219-5p	-	✓	-	HMGA2 *	[85]
	✓	✓	↓	Twist *	[61]
miR-222	✓	✓	↑	PTEN *	[87]
miR-337-3p	✓	✓	↓	PIK3CA, PIK3CB *	[100]
miR-376a	✓	✓	↑	KLF15, Caspase-8 *	[89]
miR-506	✓	✓	↑	SNAIL2 *	[62]
miR-532	✓	✓	↓	hTERT *	[86]
miR-3064					
miR-542-3p	✓	✓	↓	CDK14 *	[84]
miR-718	✓	✓	↓	VEGF *	[101]
miR-1299	✓	✓	↓	TUG1, NOTCH3 *	[68]
miR-4443	✓	-	↓	-	
miR-5159-3p					[102]

* target (connection) predicted by bioinformatics tools like TargetScan, DIANA-MicroT-CDS, miRWALK, miRDB, RNA22, PicTar, microRNA.org, PITA, miRNAnda, Starbase, etc. and/or dual-luciferase assay; ** connection predicted by expression correlation, - not part of the study; ↓ expression downregulation between compared specimens; ↑ expression upregulation between compared specimens; ✓ included in the study.

### 3.2. Long Non-Coding RNA (lncRNA) in Ovarian Cancer Progression

lncRNAs, defined as transcripts longer than 200 nucleotides without protein-coding function, also have predominantly regulatory functions and exhibit highly tissue- and organ-specific expression patterns. The main mechanisms of function include regulation of transcription by interaction with coding and non-coding RNAs, proteins, chromatin, or recruitment of transcription factors. Moreover, lncRNAs affect mRNA stability, splicing, and translation processes. By interaction with protein partners, they influence their function and localization [103,104,105,106,107]. Besides interactions with protein-coding genes or proteins, lncRNAs interact with miRNAs as competing endogenous RNA (ceRNA) or “RNA sponges” and thus impose their regulatory effect on specific targets [108]. The regulatory function of lncRNAs influences many cellular processes such as proliferation, cell growth, viability, immortality or angiogenesis, and metastatic process.

Cancer progression markers of OC, including lncRNAs, are identified mainly by comparing their expression in primary ovarian tumors and non-tumor tissues (summarized in Table 2, Table S2). Only a few studies have compared lncRNA expression profiles in primary OC tumors and metastatic tissues. The authors observed higher expression of CTD-2020K17.1 and LEF-AS1 and lower MEG3 expression in metastatic tissues of OC compared to primary tumor tissue. Knockdown of LEF1-AS1 in vitro suppressed the proliferation, migration, and invasive potential of SKOV3, OVCAR3 ovarian cancer cells [109,110,111]. The lncRNA profile, estimated by microarray analysis of expression of 4956 lncRNAs, revealed 583 upregulated and 578 downregulated lncRNAs between primary parental SKOV3 and metastatic (SKOV3.ip1EOC) sublines. The following seven lncRNAs were validated: MALAT1, H19, UCA1, CCAT1, LOC645249, LOC100128881, and LOC100292680 [112]. These lncRNAs could exert a role in OC metastatic progression. lncRNAs RP11-284N8.3.1 and AC104699.1 in the TCGA-OV dataset were differentially expressed during malignant OC progression, and functional analysis suggested their role in the tumor microenvironment and immune system [113].

In a search for new noninvasive tumor biomarkers, lncRNAs have also been studied in plasma or serum samples of OC patients. Expression of MALAT1 in plasma was significantly increased in metastatic OC patients compared with non-metastatic and healthy control samples. Moreover, OC patients with increased plasma MALAT1 had a poorer disease-free survival time [114]. lncRNA LINK-A was upregulated in OC tissue and serum samples compared to control ones. Besides, OC patients with metastases had a higher LINK-A expression in serum than patients without metastases [115]. The next study showed higher plasma LINK-A expression in OC patients with distant metastases [116]. Identification of new biomarkers in serum/plasma samples is also a part of clinical trials at present, e.g., the clinical trial NCT03738319 focused on ncRNA profile in exosomes of OC patients, last updated status showing patients recruitment [117].

The variability in genes encoding lncRNAs also plays an important role in the function of these non-coding elements. GWAS of approximately 18,000 invasive OC and 34,000 control samples of European ancestry revealed that invasive OC behavior was associated with 5294 SNPs, of which 1464 SNPs were mapped to 53 lncRNA genes [118]. Among particular genetic variants, SNP rs17427875 (minor T allele) in lncRNA HOXA11-AS1 is associated with reduced tumor growth in an in vivo mouse xenograft model based on the OC C13 cell line [119]. An association of the SNP rs920778 in HOTAIR with OC progression was reported in a case–control study of 329 OC patients and 680 controls from the Chinese population [120]. In this population, associations of other SNPs in HOTAIR (rs4759314 and 7958904) with a higher risk of OC progression were observed in 1000 OC patients and 1000 controls [121], demonstrating the potential of lncRNA genetic variability for the OC risk prediction.

The majority of studies on OC metastatic progression address the effect of lncRNAs on the EMT process through their interaction with specific EMT transcription factors and other molecules representing EMT. Association between the expression of EMT-related genes (specifically *MMP2* and *MMP9*) and lncRNAs MALAT1 (study performed on OC tumor tissue samples (*n* = 64) and reference tissue (*n* = 30)) and TP73-AS1 (study done on 60 pairs of OC tumor/control tissue) was observed. For MALAT1, it was the downregulation of *MMP2* and *MMP9*, while for TP73-AS1, the effect was opposite [65,122]. lncRNA CCAT2 was associated with EMT inhibition and regulation of expression of EMT markers, namely, E-/N-cadherin and/or vimentin [45]. Expression of lncRNA HOXA11-AS1 is associated with downregulation of expression of EMT-related markers *MMP2*, *MMP9*, *VEGF*, N-cadherin, β-catenin, and vimentin and with upregulation of E-cadherin [46]. lncRNAs ANRIL and UCA1 downregulated expression of MMPs [123,124], and lncRNA AP000695.4 regulated transcription factor *ZEB1* [125].

lncRNAs are thought to indirectly change the expression of EMT-related genes by interacting with miRNAs (as ceRNA or sponge, Table 2, Table S2) targeting these genes. For example, the above-described CCAT1 interacts with miR-152, miR-130b, and miR-490-3p, which regulate the expression of *ADAM17*, *WNT1*, *STAT3*, and *ZEB1* genes [64,126,127]. MALAT1, NEAT1, and DQ786243 interact with miR-506, a well-known interaction partner of EMT transcription factors [128,129,130] (summarized in Table 2, Table S2).

Finally, lncRNAs also seem to be involved in specific signaling pathways related to different cellular processes important for proliferation. SNHG20 affected the Wnt/β-catenin signaling pathway [131]. Other lncRNAs (MALAT1, HOXD-AS1, Linc-ROR, and HOTAIR) were associated with β-catenin inhibition [132,133,134], and, in contrast, this pathway was activated by lncRNA JPX inhibition [135]. The biologically relevant p53 pathway is regulated by lncRNA MEG3 through interaction of MEG3 with *MDM2*, leading to its suppression resulting in an increased level of p53 protein [136].

**Table 2:** lncRNAs and their implication in ovarian cancer progression.

	Experimental Model				
lncRNA	Patient Samples	Cell Lines, Xenografts	Deregulation	Target	Reference
ADAMTS9-AS2	✓	✓	↓	miR-182-5p *	[137]
ANRIL	✓	✓	↑	P15INK4b, Bcl-2 **	[138]
	✓	✓	↑	MET, MMP3 **	[123]
AOC4P	✓	✓	↓	MMP9, COL1A2 **	[139]
AP000695.4	✓	✓	↑	miR-101 **	[125]
ASAP1-IT1	✓	-	↑	-	[140]
FAM215A					
LINC00472					
BLACAT1	✓	✓	↑	miR-519d-3p *	[141]
CASC9	✓	✓	↑	miR-758-3p *	[142]
CCAT1	✓	✓	↑	miR-152, miR-130b *	[64]
	✓	✓	↑	miR-490-3p *	[126]
CCAT2	✓	✓	↑	-	[143]
	-	✓	↑	E/N-cadherin, Snail, Twist, Slug	[45]
CDKN2BAS	✓	✓	↑	GAS6 **	[144]
CTD-2020K17.1	✓	✓	↑	CARD11 *	[109]
DANCR	✓	✓	↑	miR-145	[145]
DNM3OS	✓	✓	↑	-	[146]
MEG3					
MIAT					
DQ786243	✓	✓	↑	miR-506 *	[130]
EBIC	✓	✓	↑	β-catenin, vimentin, E-cadherin **	[147]
EPB41L4A-AS2	✓	✓	↓	miR-103a *	[148]
FAM83H-AS1	✓	✓	↑	-	[149]
	✓	✓	-	HuR *	[150]
FEZF1-AS1	✓	✓	↑	miR-130a-5p *	[151]
FLVCR1-AS1	✓	✓	↑	miR-513 *	[152]
H19	-	✓	↑	miR-370-3p **	[153]
HAND2-AS1	✓	✓	↓	-	[154]
HCP5	✓	✓	↑	miR-525-5p *	[155]
HOTAIR	-	✓	↑	miR-214, miR-217 *	[156]
	✓	✓	↑	-	[157]
HOTAIRM1	✓	✓	↓	miR-106a-5p *	[158]
HOTTIP	✓	✓	↑	β-catenin **	[159]
HOXA11-AS1	✓	✓	↑	VEGF, MMP9, E-cadherin, Snail, Twist, vimentin **	[46]
HOXD-AS1	✓	✓	↑	miR-133-3p *	[133]
	✓	✓	↑	miR-186-5p *	[160]
JPX	✓	✓	↑	PI3K/AKT/mTOR pathway **	[135]
KCNQ1OT1	-	✓	↑	miR-142-5p *	[161]
	✓	✓	↑	miR-212-3p *	[162]
LEF1-AS1	✓	✓	↑	miR-1285-3p *	[110]
LINC00092	✓	✓	↑	PFKFB2 **	[163]
LINC00176	✓	✓	↑	BCL3 *	[164]
LINC00339	✓	✓	↑	miR-148a-3p *	[165]
LINC00504	✓	✓	↑	miR-1244 *, PKM2, HK2, PDK1 **	[166]
LINC00565	✓	✓	↑	cyclin D1, cyclin E1, CDK4, p16, p21 **, GAS6 *	[167]
Linc-ROR	✓	✓	↑	Wnt/β-catenin **	[134]
lncARSR	✓	✓	↑	HuR, β-catenin, ZEB1, ZEB2 **, miR-200 family *	[63]
lncRNA-ATB	-	✓	-	miR-204-3p *	[168]
	-	✓	↓	p-STAT3, E-cadherin **	[169]
LncSOX4	✓	✓	↑	-	[170]
LOC100288181	✓	✓	↑	miR-34a, miR-34c *	[171]
LOXL1-AS1	✓	✓	↑	miR-18b-5p *	[172]
LUCAT1	-	✓	↑	miR-199a-5p	[173]
MALAT1	-	✓	↑	YAP *	[174]
	✓	-	↑	-	[114]
	✓	✓	↑	-	[175]
	✓	✓	↑	PI3K/AKT pathway **	[65]
	✓	✓	↑	miR-506 **	[128]
	✓	✓	↑	MMP13, MMP19, ADAMTS1 **	[176]
	✓	✓	↑	miR-200c *	[177]
MEG3	✓	✓	↓	ATG3 **	[111]
	✓	✓	↑	PTEN **	[178]
	✓	✓	↓	miR-30e-3p	[179]
	-	✓	↓	PTEN **	[180]
	✓	✓	↓	miR-205-5p	[181]
MIF-AS1	✓	✓	↑	miR-31-5p	[182]
MIR4435-2HG	✓	✓	↑	miR-128-3p *	[183]
	✓	✓	↑	ROCK2 **	[184]
MIR4697HG	✓	✓	↑	MMP9, ERK, AKT **	[185]
NEAT1	✓	✓	↑	miR-506 *	[129]
	✓	✓	↑	miR-382-3p *	[186]
NONHSAT076754	✓	✓	↑	-	[187]
PCA3	✓	✓	↑	miR-106b-5p *	[188]
PCGEM1	✓	✓	↑	RhoA, YAP, MMP2, Bcl-xL, P70S6K **	[189]
PVT1	✓	✓	↑	EZH2 **	[190]
	✓	✓	↑	miR-133a *	[191]
RHPN1-AS1	✓	✓	↑	miR-596 *	[192]
	✓	✓	↑	miR-1299 *	[193]
SNHG3	✓	✓	↑	GSKβ/β-catenin signaling pathway **	[194]
SNHG16	✓	✓	↑	p-AKT, MMP9 **	[195]
SNHG20	✓	✓	↑	β-catenin **	[131]
SOCAR	✓	✓	↑	Wnt/β-catenin, MMP9 **	[196]
SPRY4-IT1	✓	✓	↓	E-cadherin, N-cadherin, vimentin **	[197]
TP73-AS1	✓	✓	↑	MMP2, MMP9 **	[122]
TC0101441	✓	✓	↑	KiSS1 *	[198]
TDRG1	✓	✓	↑	RhoC, R70S6K, Bcl-xL, MMP2 **	[199]
THOR	✓	✓	↑	IL-6/STAT3 **	[200]
TLR8-AS1	✓	✓	↑	TLR8, NF-κB **	[201]
TONSL-AS1	✓	✓	↑	miR-490-3p *	[202]
TPT1-AS1	✓	✓	↑	TPT1, PI3K/AKT **	[203]
TTN-AS1	✓	✓	↓	miR-15b-5p *	[204]
TUG1	✓	✓	↑	E/N-cadherin, vimentin **	[205]
UCA1	✓	✓	↑	miR-485-5p *	[124]
UNC5B-AS1	✓	✓	↑	EZH2 *	[206]
XIST	✓	✓	↑	-	[207]

* target (connection) predicted by bioinformatics tools like TargetScan, DIANA-MicroT-CDS, miRWALK, miRDB, RNA22, PicTar, microRNA.org, PITA, miRNAnda, Starbase, etc. and/or dual-luciferase assay; ** connection predicted by expression correlation, not part of the study; ↓ expression downregulation between compared specimens; ↑ expression upregulation between compared specimens; ✓ included in the study.

## 4. Interplay between Regulators ncRNA and DNA Methylation

The most typical epigenetic modifications involved in tumor transformation are DNA methylation and histone modification (specifically acetylation and methylation).

**Figure 4:**
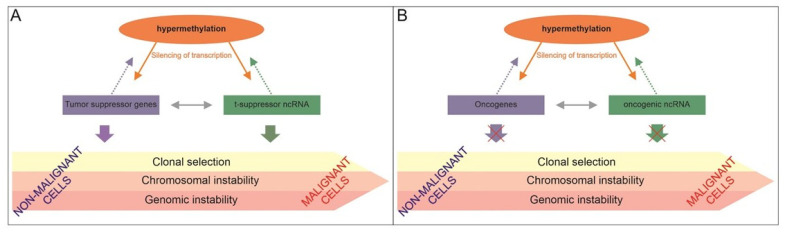
Simplified illustration of genetic and epigenetic interplay in ovary carcinogenesis. This complex process is reduced on the role of tumor suppressors (panel (**A**)) and oncogenes (panel (**B**)) in malignant transformation. Both panels indicate the effect of hypermethylation on transcription of tumor suppressor genes (**A**) and oncogenes (**B**) along with the involvement of non-coding RNAs. These may regulate tumor suppressing or oncogenic effects directly or in interaction with methylation. There are mutual regulations between non-coding RNAs and methylation status (full orange arrows) or the effect of non-coding RNAs on methylation status (dotted arrows), most likely via expression of DNA methyltransferases.

DNA methylation is a covalent modification based on attaching a methyl group to cytosine in a CG dinucleotide, often grouped into so-called CpG islands, localized predominantly in gene promoters. Hypermethylation leads to gene silencing, whereas hypomethylation is associated with disruption of genome stability. These changes are involved in the development of primary tumors and their subsequent progression. Hypermethylation and hypomethylation contribute to the development and progression of OC by altering the expression of a wide range of genes. In general, hypermethylation of genes involved in cell cycle regulation (*CDKN2A* and *CDKN2B*), DNA repair pathways (*MGMT*, *MLH1*, and *BRCA1*), and transcription factors alone (*GATA4* and *GATA5*) are important cancer-driver events. Methylation profile also interacts with other epigenetic regulatory elements such as miRNAs and lncRNAs [208,209] (Figure 4).

Expression of genes for non-coding elements is likewise affected by DNA methylation, and, vice versa, ncRNA are also indicated as regulators of this epigenetic modification.

### 4.1. miRNA-DNA Methylation Interplay

Mutual regulation of miRNA and DNA methylation in carcinogenesis was already reviewed by Wang et al. for several solid cancer types. miRNA can regulate DNA methylation by modulating the expression of DNA methyltransferases (DNMT1, DNMT3a, DNMT3b) or methylation-related proteins MeCP2 (methyl CpG binding protein 2) and MBD2/MBD4 (methyl-CpG binding domain proteins 2/4) [210]. DNA methylation is also important in miRNA biogenesis and probably stands behind the higher cancer-driver phenotype of miRNA. It was shown that regions encoding miRNA with higher DNA methylation have higher cancer-driver-related phenotype than those from unmethylated DNA regions [211].

Regarding OC, downregulation of the miR-193a-3p expression caused by gene promoter hypermethylation was reported during tumor progression with increasing methylation level from low-grade (*n* = 50) to high-grade (*n* = 46). In this study, regulation of *GRB7*, *ERBB4*, *SOS2*, and *KRAS* in the MAPK/ERK signaling pathway by miR-193a-3p was observed. Based on this study, it seems that miR-193a-3p regulates the expression of four members of the MAPK/ERK pathway, and miR-193a-3p hypermethylation led to MAPK/ERK pathway upregulation [212]. Presence of OC metastases in the lymph nodes, peritoneum, and distant organs was recorded in connection with hypermethylation of ten miRNA genes (miR-121-4, -124-3, -125B-1, -127, -129-2, -137, -193A, -203A, -339, and -375). In disseminated OC tumors, only miR-203a and miR-375 were hypermethylated. The study was conducted on 76 pairs of OC tumor/control tissues, ten pairs of tumor/peritoneal metastases, and three tumor/control/peritoneal metastases samples [213]. The miR-199a-3p, with a putative tumor suppressor function, was hypermethylated in OC tissue and OC SKOV3 cells, but not in ovarian epithelial cells IOSE386. Overexpression of this miRNA in vitro impaired tumor cell proliferation, migration, and invasiveness [214]. Other possible progression markers are miR-9-1, miR-9-3, and miR-130b. Their methylation profile associated with differentiation, tumor size, and presence of metastases of OC, as assayed for on 54 pairs of OC tumor tissue (37 patients without metastases and 17 patients with metastases) and control tissue [215]. During TGF-β1-induced EMT downregulation of TET3 in SKOV3/3AO, OC cell lines was detected. TET3 overexpression led to reverting of the TGF-β1-induced EMT phenotype by demethylation of direct downstream target miR-30d (possible EMT suppressor function) [216]. On the contrary, a negative correlation between DNMT3A/3B expression with miR-29b in 15 OC tissue samples and in vitro was found. Based on in vitro experiments (SKOV3, A2780 cell lines), a double-negative regulation between DNMT3A/3B and miR-29b, was proposed—DNMT3A/3B are downregulated by miR-29b binding to their 3′UTRs and knockdown of DNMT3A/3B lead to higher miR-29b expression through miR-29b gene promoter hypomethylation [217]. Li et al. observed suppression of miR-424-5p and miR-503-5p expression by their promoter hypermethylation in 44 OC tumors compared to ten controls and confirmed this observation also by analysis of GEO datasets (GSE14407, GSE1520, GSE38666, and GSE40595). These miRNAs probably inhibit proliferation and migration by KIF23 suppression due to direct interaction in HO8910, HO8910PM, and A2780 OC cell lines [218]. On the contrary, Let-7a-3-associated expression changes were moderately affected by DNA methylation in a cohort of 214 OC patients, and no statistically significant association with disease progression was noted [219]. Based on studies with A2780/A2780CP OC cell lines, members of the miR-200 family (miR-200b and miR-200c) are likely to modulate cell sensitivity to cisplatin by indirect interaction with DNMT1 and direct interaction with DNMT3A/3B [220]. The study on the SKOV3 cell line showed that miR-21, miR-203, and miR-205 were highly influenced by DNA methylation, as documented by demethylation treatment with 5′-aza-2′-deoxycytidine [221].

### 4.2. lncRNA-DNA Methylation Interplay

lncRNAs regulate epigenetic processes, DNA methylation, and histone acetylation as well, and are themselves strongly affected by DNA methylation. Interactions with methyltransferases/demethylases and acetyltransferases/deacetylases have been identified for several lncRNAs [105]. HOTAIR induced NF-κB in response to DNA damage and *MMP6/IL6* expression (target NF-κB genes) after platinum treatment, in vitro in a panel of sensitive (A2780, SKOV3, HEYC2, OV90, ISOE, OGROV, and OVMUNA) and resistant (A2780_CR50) OC cell lines in vitro. These results were also confirmed in the TCGA-OV dataset (higher expression in OC patients with the recurrent disease compared to primary HGSC cases) [222]. The methylation status of 67 CpG islands linked to HOTAIR was associated with poor survival of OC patients treated with platinum derivatives (six different sample sets, together 108 patients) [223]. Interaction between H19 and SAHH (*S*-adenosyl-L-homocysteine hydrolase) influenced the DNA methylation profile of several genes across the whole genome. A possible mechanism was proposed based on in vitro experiments on the HEK293 cell line. Knockdown of H19 activated SAHH, which increased methylation by DNMT3b [224].

lncRNA LOC134466 (ZFN300P1) hypermethylation was observed in 81% of serous OC tumors (from 27 samples) [225] and in cell lines SKOV3, OVCAR3, IGROV, OV90, COLO316, A2780, CaOV3, TOV112D, TOV21G, and EFO27, where the knockdown of this lncRNA reduced colony formation and proliferation. The ex vivo experiments demonstrated the feasible role of LOC134466 in attaching OC cells to the peritoneal membrane and enabling peritoneal expansion of OC metastatic lesions [226]. Based on TCGA data for 17 cancer types, epigenetic silencing of lncRNA MORT (ZNF667-AS1) in epithelial cells from 15 cancer types was extracted. Concerning OC, this was observed only for a few patients in contrast to breast, uterine, or cervix cancer patients [227]. A study focusing on the characterization of *cis*-acting lncRNA and DNA methylation showed five lncRNAs under strong methylation regulation (AC091814.2, AC141928.1, RP11-65J3.1-002, BX641110, and AF198444). Experiments on sensitive cell lines A2780/OVCAR3 and their platinum-resistant clones A2780-R/OVCAR3-R showed their possible involvement in cisplatin resistance [228].

Mutual connections and inter-regulation between ncRNA and DNA methylation are undeniable. However, it requires further clarification and mainly thorough in-depth investigation. Many potential interesting genes or DNA methylation changes have been discovered by robust high-throughput analyses and subsequently validated using in vitro and in vivo experiments. These are tools that permit us to understand the role of these targets in cell biology. The interplay between ncRNA–DNA methylation–gene expression is worthy of exploration to dissect their roles in human pathology and carcinogenesis on different levels of regulation.

## 5. Conclusions and Future Perspectives

This review addressed the involvement and mechanisms of action of non-coding RNA elements and their interplay with DNA methylation in the context of OC progression. Recognition of key players, being either lncRNAs or miRNAs, in OC progression may lead to the identification of prognostic biomarkers and formulation of novel therapeutic targets for individualized therapy.

Thanks to the advent of new robust techniques, researchers can now characterize the roles of DNA, RNA coding elements, and non-coding elements in tumor development and progression. In recent years, genomic research has brought the first useful biomarkers for OC management, e.g., patients bearing *BRCA1/BRCA2* mutations are sensitive to PARP inhibitors treatment [229]. Other promising biomarkers and tailored therapies, including the DNA repair system, are on the way (summarized in [12]). The field of ncRNA or epigenetic modifications in this setting is now open for investigations of new potential biomarkers.

The role of miRNA in OC progression is more explored than that of lncRNA. Microarray and RNA sequencing studies identified novel potential miRNAs strongly deregulated in metastasis of ovarian carcinomas compared to primary tumors, e.g., the family miR-200, which interacts with EMT-related transcription factors [44,98]. Nevertheless, despite numerous other examples, the majority of findings were not replicated, and associations scatter among different cancer types, clinical stages, or therapeutic settings. Robust clinical trials designed to test the clinical validity and utility of these candidate prognostic and predictive biomarkers are urgently needed.

Although lncRNAs are still much less explored than miRNAs, some entities were found differentially expressed in pairs of primary tumors and metastatic samples. In general, it seems that cancer progression is predominantly associated with the upregulation of lncRNA expression. Experimental studies showed connections of lncRNAs with proliferation, invasion, migration, EMT, alterations of apoptosis, and metabolic pathways (Table 2, Table S2). Recent studies focused on the identification of complex miRNA–mRNA–lncRNA interactions increased our understanding of regulatory pathways in cancer progression (as summarized in Table 1 and Table 2, Tables S1 and S2). Such integration of different regulatory levels may bring a much more solid picture of the molecular landscape of cancer and boost further efforts towards precision oncology.

DNA methylation is another effective epigenetic mechanism involved in OC progression as CpG methylation accumulates during its progression. Description of mutual interactions between miRNA/lncRNA and DNA methylation is important in the frame of the epigenetic regulation of gene expression. The advantage of DNA methylation is that its reversible character provides a promising therapeutic opportunity. This strategy is nowadays under investigation, and in some types of leukemia, demethylation therapy is already used. Uncovering these interactions may thus lead to novel therapeutic targets for a broader spectrum of cancers.

The concept of liquid biopsy, i.e., noninvasive testing using analysis of biomolecules in body fluids, mainly in serum and plasma specimens, represents the next key step to introduce experimentally identified biomarkers into clinical practice. Clinical trials focusing on the analysis of such biomarkers in body fluids are running. For example, the clinical trial NCT03738319 focuses on the expression level of lncRNA in serum samples of OC patients [117], and other clinical trials, e.g., NCT03742856, employ a multi-omics approach for tumor characterization and classification of its intrinsic heterogeneity or prediction of response to therapy [230].

On top of that, the search for biomarkers accelerated by clarifying their function using mechanistic studies based on in vitro (2D, 3D cultures, or organoids) and in vivo models (mainly mouse tumor cell xenografts or patient-derived xenografts). Patient-derived xenograft models play an essential role in the determination of a specific patient’s response to experimental therapy and the design of new drugs. Functional studies of lncRNA/miRNA interactions and their cellular functions broaden, together with in silico prediction and analysis tools, comprehension of regulatory networks in cancer progression.

We believe that additional and more robust studies on the interactive network of miRNA, lncRNA, and DNA methylation together will help to identify new prognostic and predictive biomarkers and therapeutic targets specific for OC and foster the present era of precision oncology.

## Data Availability

Please refer to suggested Data Availability Statements in section “MDPI Research Data Policies” at https://www.mdpi.com/ethics.

## Supplementary Materials

The following are available online at https://www.mdpi.com/article/10.3390/cancers13081839/s1, Supplementary Table S1: Detailed information about miRNA implication in ovarian cancer progression, Supplementary Table S2: Detailed information about lncRNA implication in ovarian cancer progression.

## Abbreviations

CAFs	cancer-associated fibroblasts
ceRNA	competing endogenous RNA
CT	computed tomography
CTCs	circulating tumor cells
DFS	disease-free survival
EMT	epithelial–mesenchymal transition
FIGO	International Federation of Gynecology and Obstetrics
GEO	Gene Expression Omnibus database
GWAS	genome wide association study
HGSC	high-grade serous carcinoma
lncRNA	long non-coding RNA
mRNA	messenger RNA
miRNA	micro-RNA
ncRNA	non-coding RNA
OC	ovarian cancer
OS	overall survival
PFS	progression-free survival
SNP	single nucleotide polymorphism
TCGA	The Cancer Genome Atlas
3′UTR	three prime untranslated region
