# Correction: Seborova et al. Non-Coding RNAs as Biomarkers of Tumor Progression and Metastatic Spread in Epithelial Ovarian Cancer. *Cancers* 2021, *13*, 1839

**DOI:** 10.3390/cancers18071136

**Published:** 2026-04-01

**Authors:** Karolina Seborova, Radka Vaclavikova, Lukas Rob, Pavel Soucek, Pavel Vodicka

**Affiliations:** 1 Toxicogenomics Unit, National Institute of Public Health, 100 42 Prague, Czech Republic; 2 Biomedical Center, Faculty of Medicine in Pilsen, Charles University, 323 00 Pilsen, Czech Republic; 3 Department of Gynecology and Obstetrics, Third Faculty of Medicine and Faculty Hospital Kralovske Vinohrady, Charles University, 100 42 Prague, Czech Republic; 4 Institute of Experimental Medicine, Czech Academy of Sciences (CAS), 142 20 Prague, Czech Republic; 5 Institute of Biology and Medical Genetics, First Faculty of Medicine, Charles University, 120 00 Prague, Czech Republic

## Text Correction

Following publication [1], the Editorial office became aware that several original articles cited in [24,67,137,150,153,159,172,175,197,203,214,217,219] were retracted by their respective journals/publishers. The authors stated that the cited papers mentioned above were retracted after publication, except for [153], which was retracted before publication and unfortunately got through the authors' check, for which the authors apologize. The authors stated that the fact does not affect the scientific conclusions.

The following corrections have been made, and the corresponding references and related content have been removed [24,67,137,150,153,159,172,175,197,203,214,217,219] to ensure scientific accuracy. Specifically,

- From Table 2, affected by retraction of articles [67,150,153,159,172,175,197,203,214,217,219], the corresponding references have been removed, and the remaining references have been re-numbered.

Following publication, the Editorial office became aware that a number of original articles cited in [24,67,137] were retracted by their respective journals/publishers.

- Section 2. Progression of Ovarian Carcinoma and Development of Metastases with affected [24]—the reference has been removed, and the remaining references have been re-numbered.
- Section 3. The Role of Non-Protein-Coding Transcripts and Their Role in the Development of Ovarian Cancer Metastasis with affected [67]—the reference has been removed, and the remaining references have been re-numbered.
- Section 3.2. Long Non-Coding RNA (lncRNA) in Ovarian Cancer Progression with affected [137]—the reference has been removed, and the remaining references have been re-numbered.

## Supplementary Material

The following corrections have been made, and the corresponding references and re-lated content have been removed [24,67,137,150,153,159,172,175,197,203,214,217,219] to ensure scientific accuracy. Specifically,

- From Table S2, affected by retraction of articles [67,150,153,159,172,175,197,203,214,217,219], with the corresponding reference references have been removed, and the remaining references have been re-numbered.

With this correction, the order of the remaining references has been adjusted accordingly. The authors state that the scientific conclusions are unaffected. This correction was approved by the Academic Editor. The original publication has also been updated.
