# Supplementary material for: Non-Coding RNAs as Biomarkers of Tumor Progression and Metastatic Spread in Epithelial Ovarian Cancer
Source: Cancers (Basel). 2021 Apr 12;13(8):1839. doi: 10.3390/cancers13081839 (PMC8069230; doi:10.3390/cancers13081839)
Supplement: Supplementary file 1 [file cancers-13-01839-s001.pdf]

Supplementary Materials

Table S1. – Detailed information about miRNAs expression and their implication in ovarian cancer progression.

miRNA	Patient samples	Cell lines, xenografts	Deregulation	Cellular function	Target	Reference
miR-9	pairs of OC tumor and adjacent control tissue (n=4)	ES—2	↓ expression in OC tissue compared to control samples	downregulation led to NF-κB overexpression, miR-9 overexpression suppresses cell growth	NF-κB*	[94]
miR-23b	EOC tissue samples (n=116) control tissue samples (n=5)	SKOV3, OVCAR3	↓ expression in EOC tissue compared to control tissue	ectopic expression inhibits proliferation and tumorigenicity, downregulation correlate with tumor aggressiveness	RUNX2*	[95]
miR-30b-3p	-	OVCAR3, IOSE80	↓ in OVCAR3 cells compared to IOSE80 cells	overexpression suppressed proliferation, promoted apoptosis, slowed cell cycle, inhibited migration and invasion	E-cadherin, β-catenin, vimentin**, CTHRC1*	[44]
miR-101	-	SKOV3	-	overexpression led to inhibition of EMT, migration and invasion	ZEB1, ZEB2*	[61]
miR-106a	pairs of OC tumor and adjacent control tissue (n=15)	SKOV3, OVCAR3	↑ expression in OC tissue compared to control tissue	inhibition suppress proliferation and invasion	PTEN**	[92]
miR-106b	OC tumor tissue samples (n=94) control tissue samples (n=17) benign tissue samples (n=13) metastases samples (n=21) borderline tumor tissue samples (n=14)	OVCAR3, HO8910PM, SKOV3/DDP, mouse xenograft	↓ expression in OC tissue and borderline tumors than non-malignant ovarian tissue and benign tumors	probably inhibits tumorigenesis and progression	RhoC**	[96]
miR-122	-	SKOV3, OVCAR3, mouse xenograft	-	suppress EMT	P4HA1*	[77]

miR-138	OC tumor tissue samples (n=78)	SKOV3, TOV-112D, A1847, mouse xenograft	↓ expression in invasive cells	expression inhibits OC metastasis to other organs	SOX4, HIF-1α*	[97]
miR-141	OC tumor tissue samples (n=49) control tissue samples (n=12)	SKOV3, OVCA433, A2780cp, HOSE 96-9-10, HOSE11-12, HOSE 17-1, mouse xenograft	↑ expression in OC tissue compared to control tissue	knockdown led to inhibition of proliferation, anoikis resistance, tumor growth and peritoneal metastasis	KLF12*	[98]
miR-145	HGSC tissue samples (n=48) control tissue samples (n=19)	HO8910, HO8910PM, OVCAR3, HEK293T, FTE187, HEY, A2780, mouse xenograft	↓ expression in OC tissue compared to fimbria	overexpression suppresses proliferation, migration and invasion *in vitro* and inhibits tumor growth and metastasis *in vivo*	MTDH*	[93]
miR-193b	pairs of HGSC omental metastases and adjacent control omentum (n=7)	Skov3ip1, HeyA8, ES2, mouse xenograft	↓ expression in OC lines, in omental metastases compared to normal omentum	downregulation caused by microenvironment through DNMT1	uPA*	[31]
miR-199a-5p	-	HO-8910, ES-2, FTE187	↓ expression in OC cell lines compared to control cell line	exogenous expression inhibits proliferation, overexpression inhibit invasion *in vitro*	NF-κB1*	[99]
miR-200a	OC tumor and control tissue (n=57)	OVCAR3, A2780, HOSEpiC, HEK293T	↑ expression in tumor tissue and cell lines compared to control tissue and cell line	enhances migration and invasion, expression associated with lymph node metastases	PTEN*	[75]
miR-200c	-	SKOV3	↓ expression in CD117+CD44+ CSCs from SKOV3 cell line	overexpression led to EMT inhibition	ZEB1, vimentin, E-cadherin**	[45]
	-	SKOV3	-	overexpression reduces invasion ability of cells *in vitro*, *in vivo* decrease tumorgenicity, overexpression decreases expression of HOTAIR, Snail and increase E-cadherin *in vitro*/*in vivo*	HOTAIR, Snail, E-caherin**	[100]
miR-204	dataset (TCGA-OV)	SKOV3, mouse xenograft	somatic loss	possible suppressor of tumor growth and metastasis, loss led to activation AKT/mTOR signaling	BDNF*	[101]

miR-205	OC tumor tissue samples (n=110) control tissue samples (n=3)	HO-8910, SKOV3, HO-8910PM, SKOV3ip, SKOV3/DDP, COC1, mouse xenograft	↑ expression in OC tissue compared to control tissue	ectopic expression led to enhanced proliferation, migration, invasion, *in vivo* expression promoted the growth and metastasis of tumor	SMAD4, PTEN*	[90]
miR-219-5p	-	SKOV3, mouse xenografts	-	overexpression led to suppression of proliferation, invasion and migration	HMGA2*	[87]
miR-219-5p	pairs of EOC tissue and adjacent control tissue (n=20)	SKOV3, OVCAR3, CAOV3, A2780	↓ expression in tumor tissue compared to adjacent control tissue	overexpression led to inhibition of progression, Wnt/β-catenin signaling pathway	Twist*	[62]
miR-222	pairs of OC tumor and adjacent control tissue (n=40)	SKOV3, OVCAR3, A2780, HOSEpiC	↑ expression in OC tissue compared to control cervical tissue and cell lines	overexpression led to enhanced migration and invasion	PTEN*	[89]
miR-337-3p	EOC tissue samples (n=105) control tissue samples (n=51)	HEK293T, A2780, SKOV3, OVCAR3, ES-2, OV-90, CAOV3, HOSEpiC, mouse xenograft	↓ expression in EOC tissue compared to control tissue	ectopic expression inhibits proliferation, induces apoptosis, cell cycle arrest in G0/G1 phase *in vitro*, through interaction with PIK3CA/B reducing activity of PI3K/Akt signaling pathway, *in vivo* miR-337-3p acts as TSG	PIK3CA, PIK3CB*	[102]
miR-376a	OC tumor tissue samples (n=32) control tissue samples (n=10)	SKOV3, A2780, HO8910, HO8910PM, mouse xenograft	↑ expression in OC tissue compared to adjacent tissue, higher expression in high metastatic HO8910PM compared to less metastatic HO8910 OC cell line	overexpression stimulates the proliferation, migration and invasion, *in vivo* showed role in cancer progression	KLF15, Caspase-8*	[91]
miR-506	EOC tissue samples (n=204)	mouse xenograft	↑ expression in early stages vs. late stages	regulates E-cadherin, vimentin, N-cadherin in the suppression of EMT/metastasis	SNAIL2*	[63]
miR-532 miR-3064	EOC tissue samples (n=60)	SKOV3, ES-2, NOEC, mouse xenograft	↓ expression in OC tissue compared to	overexpression suppresses the proliferation, EMT and	hTERT*	[88]

	control tissue samples (n=20)		control ovarian tissue and cell lines	invasion, *in vivo* overexpression inhibits the growth of OC cells		
miR-542-3p	EOC tissue samples (n=28) control tissue samples (n=12)	OVCAR3, SKOV3, HO8910, HOSEpiC	↓ expression in tumor tissue and cell lines vs. normal human ovarian epithelial cell line	overexpression suppresses the proliferation of OC cells *in vitro*/ *in vivo*, migration and invasion *in vitro*	CDK14*	[86]
miR-718	pairs of OC tumor and adjacent control tissue (n=20)	ES-2, SKOV3, CAOV3, OVCAR3	↓ expression in OC tissue than in non-malignant ovarian tissue, lower expression also in cell lines	expression led to inhibition of proliferation in *vitro*/*in vivo*	VEGF*	[103]
miR-1299	EOC tissue samples (n=35 control tissue samples (n=16)	A2780, CAOV3, SKOV3	↓ expression in OC tissue compared to control samples	expression correlates with tumor differentiation, overexpression inhibits proliferation, colony formation, cell arrest in G0/G1 arrest *in vitro*, *in vivo* overexpression suppresses tumor growth	TUG1, NOTCH3*	[70]
miR-4443 miR-5159-3p	tumor tissue samples (serous n=31, endometroid n=8, mucinous n=6) control tissue (n=45)	-	↓ expression in OC tissue compared to controls, higher downregulation in metastatic samples	-	-	[104]

* target (connection) predicted by bioinformatics tools like - TargetScan, DIANA-MicroT-CDS, miRWALK, miRDB, RNA22, PicTar, microRNA.org, PITA, miRNAnda, Starbase etc. and/or dual-luciferase assay; ** connection predicted by expression correlation; - not part of the study.

Table S2. – Detailed information about lncRNAs expression and their implication in ovarian cancer progression.

lncRNA	Patient samples	Cell lines, xenografts	Aberration	Cellular function	Target	Reference
ADAMTS9-AS2	OC tumor tissue samples (n=47) control tissue samples (n=?)	SKOV3, HO8910, A2780, OVCAR, HOSEpiC, mouse xenograft	↓ expression in OC tissue compared to control tissue and cell lines	reduces proliferation, invasion, migration, EMT, restrained tumor growth *in vivo*	miR-182-5p*	[140]
ANRIL	EOC tumor tissue samples (n=102) control tissue samples (n=30)	SKOV3, OVCAR3, A2780, Hey, OVCA429, OVCA433, mouse xenograft	↑ expression in OC tissue compared to noncancerous tissue	promotes proliferation (cell cycle progression, apoptosis and senescence inhibition), downregulation of P15INK4b and upregulation Bcl-2	P15INK4b, Bcl-2**	[141]
	HGSC tumor tissue samples (n=68) control tissue samples (n=30)	SKOV3, HO8910, SKO3.ip1, HO8910-PM	↑ expression in OC tissue compared to noncancerous tissue	expression higher in metastatic cell lines, silencing impaired migration and invasion	MET, MMP3**	[125]
AOC4P	EOC tumor tissue samples (n=70) control tissue samples (n=10)	HEY-A8, HEY, HO8910-PM, HO8910, SKOV3-IP, SKOV3	↓ expression in OC cell lines and tissue compared to controls	knockdown in poorly metastatic cell lines enhances migration, invasion, overexpression in highly metastatic cell lines reduces metastatic capabilities	MMP9, COL1A2**	[142]
AP000695.4	TCGA-OV data	SKOV3, A2780, OVCAR3, mouse xenograft	↑ expression in mesenchymal subtype vs. epithelial subtype	expression positively correlates with *ZEB1* expression, knockdown reduced tumorigenicity and metastasis *in vivo*	miR-101**	[127]
ASAP1-IT1 FAM215A LINC00472	EOC tumor tissue samples (n=266)	-	↑ expression in OC tissue	higher expression associated with favorable OS, maybe involved in OC progression	-	[143]

BLACAT1	OC tumor tissue samples (n=30) control tissue samples (n=29)	SKOV3, mouse xenograft	↑ expression in OC tissue compared to control tissue	knockdown inhibits proliferation, migration, invasion, *in vivo* inhibits tumor growth	miR-519d-3p*	[144]
CASC9	pairs of OC tumor and control tissue (n=43)	SKOV3, CAOV3, OV420, A2780, ES-2), mouse xenograft	↑ expression in OC tissue compared to control tissue and cell lines	promotes proliferation, migration, invasion, *in vivo* accelerates tumor growth	miR-758-3p*	[145]
CCAT1	pairs of EOC tumor and control tissue (n=72)	HO8910, HO8910PM, OVCAR3, SKOV3, CAOV3	↑ expression in OC tissue compared to adjacent non-tumor tissue	CCAT1 regulates miR-152/miR-130b (their targets are *ADAM17, WNT1, STAT3, ZEB1*) - their knockdown inhibits EMT	miR-152, miR-130b*	[65]
	pairs of EOC tumor and control tissue (n=25)	SKOV3, CAOV3	↑ expression in OC tissue compared to control tissue	expression stimulated by TGF-β1, knockdown decreased migration, invasion and downregulated expression EMT-related markers	miR-490-3p*	[128]
CCAT2	OC tumor tissue samples (n=109) control tissue samples (n=45)	SKOV3, IGROV1, A2780, OVCAR3	↑ expression in OC tissue compared to control tissue and cell lines	expression correlates with presence of distant metastasis, knockdown suppresses proliferation, migration and invasion	-	[146]
	-	SKOV3, A2780, HO8910, HOSE-HUM-CELL-0088	↑ expression in OC cell lines compared to controls	knockdown inhibits EMT, migration and invasion	E/N-cadherin, Snail, Twist, Slug	[46]
CDKN2BAS	OC tumor tissue samples (n=44) control tissue samples (n=16)	A2780, HO8910, HEY, SKOV3, IOSE80	↑ expression in OC tissue compared to control tissue	overexpression enhances proliferation, migration	GAS6**	[147]
CTD-2020K17.1	pairs of HGSC primary tumors and omental metastasis (n=38)	SKOV3, OVCAR3, CAOV3	↑ expression in omental metastases tissue compared to primary tumors	overexpression promotes migration, invasion, proliferation	CARD11*	[111]

DANCR	pairs of OC malignant tumor and control tissue (n=20)	A2780, PA-1, SKOV3, HO8910, HOSEpic, mouse xenograft	↑ expression in OC cell lines and malignant tumor tissue compared to controls	knockdown impairs tumor growth through angiogenesis inhibition	miR-145	[148]
DNM3OS MEG3 MIAT	TCGA-OV data	SKOV3	↑ expression in OC tissue	pathway analysis revealed connection with EMT pathway, especially for DNM3OS	-	[149]
DQ786243	pairs of OC tumor and control tissue (n=30)	SKOV3, OVCAR3, PEO1, A 2780, mouse xenograft	↑ expression in OC tissue compared to control tissue and cell lines	knockdown inhibits proliferation, invasion, migration, colony formation, *in vivo* inhibits tumor growth	miR-506*	[132]
DSCR8	-	IOSE80, A2780, SKOV3, OVCAR3, PEO1	↑ expression in OC cell lines compared to controls	inhibition suppresses proliferation	miR-3192-5p/YY1*	[150]
EBIC	pairs of OC tumor and control tissue (n=126)	OVCA429, SKOV3	↑ expression in OC tissue compared to control tissue	siRNA-EBIC transfection inhibits proliferation, invasion, migration, downregulate expression of β-catenin, vimentin, c-myc and upregulation of E-cadherin	β-catenin, vimentin. E-cadherin**	[151]
EPB41L4A-AS2	datasets (GSE83693, GSE18520), pairs of OC tumor and control tissue (n=126)	HO8910, OV-90, OVCAR3, SKOV3, HOSEpiC, mouse xenograft	↓ expression in OC tissue and cells compared to controls	overexpression inhibits proliferation, migration, colony formation and invasion, *in vivo* represses tumor formation	miR-103a*	[152]
FAL1	-	SKOV3, HO8910PM, mouse xenograft	↑ expression in cells	higher expression in tumor derived exosomes enhances migration, invasion, metastasis, *in vivo* tumors in mice larger and heavier	PTEN, Akt**	[153]

FAM83H-AS1	pairs of OC tumor and control tissue (n=100)	HOSE6.3, OVCAR3	↑ expression in OC cell lines and tumor tissue compared to controls	expression correlates with distant metastases, downregulation inhibits proliferation and invasion	-	[154]
	pairs of OC tumor and control tissue (n=80) (44 metastatic samples)	ES-2, SKOV3, A2780, SW626, IOSE386	-	inductive effect on metastasis	HuR*	[155]
FEZF1-AS1	pairs of EOC tumor and control tissue (n=52)	PEO1, SKOV3, COC1, CAOV3, A2780, 3AO, IOSE80	↑ expression in OC cell lines and tissue compared to controls	silencing suppresses migration, proliferation, invasion, colony formation, enhances apoptosis	miR-130a-5p*	[156]
FLVCR1-AS1	OC serous tumor tissue, control tissue and serum samples (n=50)	A2780, 3AO, PEOI, SKOV3, OVCAR3, OVCAR8	↑ expression in OC cell lines, tumor tissue and serums samples compared to controls	downregulation inhibits cell growth, migration, invasion and EMT	miR-513*	[157]
H19	-	SKOV3, OVCAR3	↑ expression in OC cells	TGF-β upregulates H19 and downregulates miR-370-3p, H19 knockdown/miR-370-3p overexpression suppresses EMT	miR-370-3p**	[158]
HAL	pairs of serous tumor and adjacent tissue (n=30)	SKOV3, OVCAR3, A2780, mouse xenograft	↓ expression in OC tissue and cell lines	overexpression inhibits proliferation, migration, invasion, promoted apoptosis, downregulates Twist1 expression, *in vivo* inhibits tumorgenicity via EMT inhibition	Twist1**	[159]
HAND2-AS1	datasets (GSE69428, TCGA-OV)	SKOV3, FT-194, PEA1, PEA2, PEO14, PEO23, OVSAMO, KURAMOCHI, HEyA8, TOV21G	↓ expression in OC lines compared to control cell line	downregulation cause by hypermethylation, HAND2-AS1 acts tumor suppressor gene	-	[160]
HCP5	pairs of OC tumor and control tissue (n=44)	SKOV3, OVCA433, HOSE11-12, mouse xenograft	↑ expression in OC cell lines and tissue compared to controls	silencing decreases proliferation, invasion, migration, EMT process,	miR-525-5p*	[161]

HOTAIR	-	SKOV3, OVCAR3, A2780	↑ expression in SKOV3, OVCAR3 compared to A2780	activates Wnt/β-catenin pathway silencing inhibits proliferation, migration, invasion, interaction with *PIK3R3* via miR-214 and miR-217	miR-214, miR-217*	[162]
	EOC tumor tissue samples (n=64) control tissue samples (n=29)	SKOV3.ip1, HO8910-PM, HEY-A8	↑ expression in OC tissue compared to control tissue	suppression reduced migration/invasion in highly metastatic cell lines	-	[163]
HOTAIRM1	OC tumor tissue samples (n=68) control tissue samples (n=48)	SKOV3, OVCAR3, A2780, ES-2, HOSEpiC, mouse xenograft	↓ expression in OC cell lines and tissue compared to controls	overexpression suppresses proliferation, invasion, promoted apoptosis	miR-106a-5p*	[164]
HOTTIP	pairs of OC tumor and control tissue (n=69)	SKOV3, A2780, OVCAR3	↑ expression in OC tissue compared to control tissue	knockdown decreased proliferation, invasion	β-catenin**	[165]
HOXA11-AS1	HGSC tumor tissue samples (n=129) control tissue samples (n=38)	SKOV3, OVCAR3, A2780, OVCA433, OVCA429, TOV112D	↑ expression in OC tissue compared to noncancerous tissue	overexpression enhances proliferation, invasion, migration, expression associated with expression of *VEGF*, *MMP9*, β-catenin, E-cadherin, Snail, Twist, vimentin	VEGF, MMP9, E-cadherin, Snail, Twist, vimentin**	[47]
HOXD-AS1	pairs of EOC tumor and control tissue (n=43)	SKOV3, HO8910, ES-2, CAOV3	↑ expression in OC tissue compared to control tissue and cell lines	promotes proliferation, invasion, EMT via activating Wnt/β-catenin signaling pathway	miR-133-3p*	[135]
	EOC tumor tissue samples (n=36) control tissue samples (n=14)	A2780, SKOV3	↑ expression in OC tissue compared to control tissue	inhibition reduces migration, invasion, EMT, interaction with miR-186-5p downregulates *PIK3R3*	miR-186-5p*	[166]
JPX	pairs of OC tumors and adjacent nontumor tissue (n=32)	OVCAR3	↑ expression in OC tissue compared to para-carcinoma tissue and cell lines	possible activation of PI3K/AKT/mTOR pathway, which led to proliferation,	PI3K/AKT/ mTOR pathway**	[138]

				invasion and migration of cells		
KCNQ1OT1	-	SKOV3, OVCAR3, IOSE80	↑ expression in OC cells compared to control	enhances proliferation, migration	miR-142-5p*	[167]
	pairs of EOC tumor and control tissue (n=174)	IOSE80, OVCAR3, SKOV3, A2780, OV90	↑ expression in OC cell lines and tumor tissue compared to controls	overexpression enhances cell growth, migration, invasion	miR-212-3p*	[168]
LEF1-AS1	OC tumor tissue (n=62) (metastatic (n=28), non-metastatic (n=34))	IOSE80, SKOV3, OVCAR3, OVCAR5, A2780	↑ expression in metastatic tissue compared to non-metastatic	knockdown suppresses proliferation, migration, invasion	miR-1285-3p*	[112]
LINC00092	serous tumor tissue samples (n=58) control tissue samples (n=25) TCGA-OV	SKOV3, A2780, mouse xenograft	↑ expression in OC cells, OC patients with metastases compared to OC patients without metastasis and control samples	expression induced by CAF-secreted CXCL14, promotes cancer progression by altering a glycolysis	PFKFB2**	[169]
LINC00176	GSE38666, pairs of OC tumor tissue and control tissue (n=56)	CAOV3, 3AO, SKOV3, HO8910, A2780, CHO 1-15, mouse xenograft	↑ expression in OC cell lines and tissue compared to controls	silencing promotes proliferation, migration, invasion, upregulates *CP* expression through *BCL3*	BCL3*	[170]
LINC00339	pairs of OC tumor tissue and control tissue (n=75)	SKOV3, A2780, OVCAR3, HO-8910, HOSEpiC, mouse xenograft	↑ expression in OC tissue compared to control	higher expression associates with proliferation, migration and invasion, *in vivo* promotes tumor growth	miR-148a-3p*	[171]
LINC00460	pairs of EOC tumor and control tissue (n=98)	SKOV3, A2780, OVCAR, HO8910	↑ expression in OC tissue compared to control tissue and cell lines	knockdown suppresses proliferation, migration, invasion; higher expression associated with lymph node metastasis	miR-338-3p*	[172]
LINC00504	pairs of OC tumor and adjacent tissue (n=45)	HOSEpiC, A2780, CAOV3, HO8910, OVCAR3, SKOV3	↑ expression upregulation in OC	knockdown inhibits proliferation, enhance apoptosis, decreases	miR-1244*, PKM2, HK2, PDK1**	[173]

			tissue compared to control	glycolysis-related genes expression (*PKM2*, *HK2*, *PDK1*)		
LINC00565	datasets (TCGA, GSE26193, GSE52037, GSE38666, GSE40595)	OVCAR3, SKOV3, HO8910, A2780, HEY, IOSE, mouse xenograft	↑ expression in OC tissue compared to control tissue	knockdown inhibits proliferation, invasion, migration, *in vivo* inhibits tumor growth	cyclin D1, cyclin E1, CDK4, p16, p21**, GAS6*	[174]
LINC00963	OC tumor tissue samples (n=35) control tissue samples (n=35)	A2780, TOV112D, OVCAR3, SKOV3, IOSE80), mouse xenograft	↑ expression in OC tissue compared to control	downregulation inhibits migration, invasion, invert EMT triggered by TGF-β1, represses tumorgenicity *in vivo*	miR-378g	[175]
Linc-ROR	HGSC tumor tissue samples (n=39) control tissue samples (n=20) fallopian tube tissue (n=20)	SKOV3, A2780, mouse xenograft	↑ expression in OC tissue compared tocontrol tissue and normal fallopian tube tissue	promotes proliferation, migration, invasion, knockdown inhibits EMT via repression of Wnt/β-catenin pathway	Wnt/β-catenin**	[136]
lncARSR	pairs of EOC tumor and adjacent tissue (n=76)	SKOV3, HO8910, ES-2, CAOV3, IOSE80	↑ expression in OC tissue compared to control tissue and cell lines	higher expression promotes proliferation, invasion and associate with lymph node metastasis	HuR, β-catenin, ZEB1, ZEB2**, miR-200 family*	[64]
lncRNA-ATB	-	SKOV3, A2780, 293T	-	downregulation inhibits proliferation, induces apoptosis	miR-204-3p*	[176]
	-	SKOV3, HOSEpic	↓ expression in OC cells compared to controls	downregulation led to reduce proliferation, invasion, migration, promoted apoptosis	p-STAT3, E-cadherin**	[177]
LncSOX4	EOC tumor tissue samples (n=30) control tissue samples (n=18)	SKOV3, HO8910-PM, OVCAR3, IOSE-80	↑ expression in OC cell lines and tumor tissue compared to controls	silencing impaired proliferation, expression associated with distant metastasis	-	[178]

LOC100288181	datasets (GSE3668, GSE18520, GSE9891, GSE26193, GSE63885)	HEY-T30, SKOV3, mouse xenograft	↑ expression in OC tissue compared to normal tissue	knockdown suppresses proliferation, colony formation, invasion, migration, *in vivo* inhibits tumorigenicity	miR-34a, miR-34c*	[179]
LOXL1-AS1	pairs of OC tumor and control tissue (n=45)	A2780, SKOV3, CAOV3, OVCAR3, IOSE80	↑expression in OC tissue compared to controls	knockdown inhibits growth, aggressive phenotype, through interacting with miR-18b-5p regulate progression and metastasis	miR-18b-5p*	[180]
LUCAT1	-	CAOV3, SKOV3, HO8910, IOSE80	↑ expression in cells	knockdown decreases proliferation and colony formation	miR-199a-5p	[181]
MALAT1	-	SKOV3, SKOV3-CR	↑ expression in non-adherent spheres formed by adherent OC cells	knockdown reduces cell stemness, decreases sphere forming ability	YAP*	[182]
	plasma from EOC patient with distant metastases (n=47), EOC patients without metastases (n=47), control samples (n=47)	-	↑ expression in OC patients with distant metastases compared patients without metastasis and healthy controls	higher expression associated with poorer DFS, possible independent predictor of survival	-	[116]
	serum samples from EOC patients (n=60), control serum samples (n=?)	SKO3.ip1, HO8910.PM, SKOV3, HO8910, HUVEC mouse xenograft	↑ expression in metastatic OC cells compared to OC cells, expression in serum upregulated compared to control samples	elevated serum exosomal expression correlated with metastatic phenotype	-	[183]
	EOC tumor tissue samples (n=64) control tissue samples (n=30)	SKOV3, OVCAR3, HO8910, A2780, mouse xenograft	↑ expression in OC tissue compared to control tissue and cell lines	inhibition of MALAT1 impeded proliferation, invasion, metastasis, downregulation EMT-related genes and MMPs	PI3K/AKT pathway**	[66]

	pairs of OC tumors and adjacent nontumor tissue (n=30)	SKOV3, A2780, HO8910, CAOV3	↑ expression in OC tissue compared to control tissue and cell lines	promotes tumor growth, knockdown inhibits proliferation and DNA synthesis	miR-506**	[130]
	EOC tumor tissue samples (n=45) control tissue samples (n=37)	OVCAR3, SKOV3	↑ expression in OC cell lines and tumor tissue compared to controls	overexpression enhances proliferation, migration and invasion	MMP13, MMP19, ADAMTS1**	[184]
	pairs of OC tumor and control tissue (n=50)	OVCAR3, CAOVš, SKOV3, PA-1, MES-OV, UWB1.289m OV-90, HEY-T30, HOSEpic	↑ expression in OC cell lines and tumor tissue compared to controls	knockdown suppresses proliferation, viability, migration and invasion	miR-200c*	[185]
MEG3	benign tumor tissue samples (n=8), control tissue samples (n=17), borderline tissue samples (n=6), primary EOC carcinoma tissue (n=95), omental metastasis samples (n=25)	OVCAR3, A2780, mouse xenograft	↓ expression in OC tissue, especially in omentum tumors compared to control tissue and benign tumors	upregulation inhibits proliferation, formation, promoted apoptosis, *in vivo* suppress tumorigenesis	ATG3**	[113]
	EOC tumor tissue samples (n=90)	HOSE, COV318, HEY, PEO1, mouse xenograft	↑ expression in OC tissue associate with better PFS, OS	overexpression inhibits migration, invasion, inhibits spheroid growth in extracellular matrix, *in vivo* inhibits tumor growth	PTEN**	[186]
	OC tumor tissue samples (n=30) control tissue samples (n=10) datasets (GSE29450, GSE54388)	SKOV3, OVCAR3, CAOV4, IOSE80, HEK293T, HOSEpic	↓ expression in OC tissue compared to normal tissue and cell lines together with LAMA4 and downregulation of miR-30e-3p	overexpression enhances *LAMA4* expression by sponging miR-30e-3p	miR-30e-3p	[187]

	-	SKOV3, CAOV3, OVCAR3, mouse xenograft	↓ expression in OC cells	enhanced expression of *PTEN* suppress proliferation, invasion, migration	PTEN**	[188]
	pairs of OC tumor tissue and adjacent tissue (n=20)	SKOV3, OVCAR3, OVCAR5, OVCAR8	↓ expression in OC tissue compared to control tissue and cell lines	MEG3 overexpression and miR-205-5p knockdown inhibit viability, migration, invasion and promoted apoptosis	miR-205-5p	[189]
MIF-AS1	pairs of OC tumor tissue and control tissue (n=50)	IOSE80, OC3, HO8910, ES-2, SKOV3	↑ expression in OC cell lines and tissue compared to controls	knockdown decreases proliferation, migration, invasion	miR-31-5p	[190]
MIR4435-2HG	pairs of OC tumor and adjacent tissue (n=42)	SKOV3, CAOV3, A2780, OVCAR3, IOSE80, HEK293T	↑ expression in OC cell lines and tissue compared to controls	knockdown inhibits proliferation, invasion, migration, induces apoptosis via miR-128-3p/*CDK14* axis	miR-128-3p*	[191]
	pairs of OC tumor and adjacent tissue (n=63)	UWB1.289	↑ expression in OC tissue	overexpression together with *ROCK2* promote proliferation, inhibits apoptosis	ROCK2**	[192]
MIR4697HG	pairs of OC tumor and control tissue (n=15)	SKOV3, OVCAR3, CAOV3, CoC1, mouse xenograft	↑ OC tissue compared to control tissue, OC cell lines (OVCAR3, SKOV3)	knockdown inhibits proliferation, colony formation, downregulation of *MMP9, ERK, AKT*	MMP9, ERK, AKT**	[193]
NEAT1	HGSC tumor tissue samples (n=75), control tissue samples (n=75)	A2780, HO8910, SKOV3, OVCAR3, CAOV3, ES-2, OV420, mouse xenograft	↑ expression in OC tissue compared to control tissue	knockdown inhibits proliferation, invasion, *in vivo* inhibit tumor growth, stability enhanced by LIN28B	miR-506*	[131]
	pairs of OC tumor and control tissue (n=67)	ES-2, SKOV3	↑ expression in OC tissue compared to para-tumor tissue and cell lines	regulating miR-382-3p/*ROCK1* in the metastastic process	miR-382-3p*	[194]

NONHSAT076754	EOC tumor tissue samples (n=70) control tissue samples (n=10)	SKOV3, OVCAR5, OVCAR3, OVCAR8, HO8910, HOSEpic, mouse xenograft	↑ expression in OC tissue and cells compared to controls	knockdown inhibits migration, invasion, *in vivo* depletion reduces EOC metastasis	-	[195]
PCA3	tumor tissue samples (n=29) control tissue samples (n=7)	OVCAR3, A2780	↑ expression in OC tissue compared to control tissue and cell lines	knockdown led to inhibition of proliferation, migration, invasion, downregulation of expression - RhoC, Bcl/xl, MMP2, P70S6K	miR-106b-5p*	[196]
PCAT-1	pairs of OC tumor and adjacent tissue (n=20)	A2780, SKOV3	↑ expression in OC tissue compared to normal tissue	expression silencing reduces proliferation, migration, invasion, increase apoptosis, knockdown repress expression of cyclin D1, CDK3, p53, BAX, cleaved caspase 3, vimentin with restore miR-124-3p expression	Cyclin D1, CDK3, p53, BAX, vimentin, miR-124-5p**	[197]
PCGEM1	EOC tumor tissue samples (n=50) control tissue samples (n=14)	A2780, OVCAR3, mouse xenograft	↑ expression in OC cell lines and tumor tissue compared to controls	upregulation induces proliferation, migration and invasion	RhoA, YAP, MMP2, Bcl-xL, P70S6K**	[198]
PTAF	datasets (TCGA-OV, GSE9891)	SKOV3, A2780, OVCAR3, mouse xenograft	-	silencing inhibits tumor progression and metastasis *in vivo*	miR-25, SNAIL2**	[67]
PVT1	EOC tumor tissue samples (n=231) control tissue samples (n=58)	SKOV3, HO8910, ES-2, SW626, A2780	↑ expression in OC tissue compared to control tissue and cell lines	knockdown impaired proliferation, migration, invasion, repression miR-214 via *EZH2*	EZH2**	[199]
	pairs of OC tumor and control tissue (n=42)	HEY, SKOV3, OVCAR3	↑ expression in OC tissue compared to control tissue	knockdown inhibits proliferation, migration, invasion	miR-133a*	[200]
RHPN1-AS1	pairs of EOC tumor and adjacent tissue (n=86)	CAOV3, ES-2, A2780, OV-90, OVCAR3	↑ expression associate with poor prognosis	upregulation promotes proliferation and	miR-596*	[201]

				metastasis, activation miR-596/LETM1/FAK-PI3K/Akt signaling pathway		
	pairs of OC tumor tissue and control tissue (n=57)	OVCAR5, OVCAR3, A2780, SKOV3, IOSE80	↑ expression in OC tissue compared to controls	knockdown inhibits proliferation, migration, invasion and promotes miR-1299 expression	miR-1299*	[202]
SNHG1	pairs of EOC tumor and para-carcinoma tissue (n=20)	IOSE25, CAOV3, SKOV3, ES-2, A2780	↑ expression in OC cell lines and tumor tissue compared to controls	knockdown inhibits proliferation, clone formation, invasion, metastasis, promoted apoptosis	MMP2, MMP9**	[203]
SNHG3	pairs of EOC primary tumors and adjacent control tissue (n=76)	OVCAR3, A2780, SKOV3, ES-2	↑ expression in OC tissue compared to control tissue and cell lines	knockdown inhibits proliferation, invasion abilities, downregulation of Cyclin D1, CDK1, MMP9, MMP3	GSKβ/β-catenin signaling pathway**	[204]
SNHG16	pairs of EOC tumor and control tissue (n=103)	SKOV3, ES2, HOP8910, OMC685, IOSE-29	↑ expression in OC cell lines and tumor tissue compared to controls	higher expression associated with distant metastasis, knockdown decrease proliferation, invasion and migration	p-AKT, MMP9**	[205]
SNHG20	pairs of OC primary tumors and adjacent control tissue (n=16)	SKOV3, OVCA429, OVCA433, OVCAR3, HOSE	↑ expression in OC tissue compared to non-tumorous tissue	knockdown suppresses the ovarian cancer progression	β-catenin**	[133]
SOCAR	pairs of HGSC primary tumors and omental metastases (n=50)	SKOV3, OVCAR3, CAOV3, HO89PM	↑ expression in OC tissue compared to control	higher expression associates with progression, overexpression promotes proliferation, migration and invasion	Wnt/β-catenin, MMP9**	[206]
SPRY4-IT1	pairs of OC tumor and control tissue (n=15)	SKOV3, HO8910, ES-2, CAOV3, IOSE80	↓ expression in OC tissue compared to controls	overexpression reduces proliferation, colony formation, migration, invasion, promoted apoptosis, arrest cell cycle	E-cadherin, N-cadherin, vimentin**	[207]

TP73-AS1	pairs of OC tumor and control tissue (n=60)	OVCA429, OVCA433, SKOV3	↑ expression in OC tissue compared control tissue and cell lines	knockdown suppresses proliferation, invasion, migration, overexpression enhanced expression of MMP2 and MMP9	MMP2, MMP9**	[124]
TC0101441	EOC tumor tissue samples (n=74) control tissue samples (n=20)	SKOV3, CAOV3, OVCAR3, PEO1, PEO4	↑ expression in OC tissue	*in vitro/in vivo* loss-of function assay promotes invasive and metastatic capabilities	KiSS1*	[208]
TDRG1	EOC tumor tissue samples (n=95), control tissue samples (n=26)	OVCAR3, A2780	↑ expression in OC cell lines and tumor tissue compared to controls	knockdown suppresses proliferation, migration and invasion	RhoC, R70S6K, Bcl-xL, MMP2**	[209]
THOR	pairs of OC tumor tissue and control tissue (n=90)	SKOV3, A2780, OVCA429, 3AO, PEO-1, HO8910	↑ expression in OC tissue compared to control tissue	knockdown inhibits growth, metastasis, self-renewal of OC cells, drives cell progression via IL-6/STAT3	IL-6/STAT3**	[210]
TLR8-AS1	OC tumor tissue samples (n=158), datasets (TCGA, GSE82059)	OV90, SKOV3, mouse xenograft	↑ expression in OC cell lines and tissue compared to controls	TLR8-AS1 is regulated by CAFs, augment metastasis and chemoresistance, upregulates TLR8, activate NF-κB	TLR8, NF-κB**	[211]
TONSL-AS1	pairs of EOC tumor and adjacent tissue (n=62)	OVCAR3	↑ expression in OC tissue compared to control	through interaction with *CDK1* influence cell proliferation	miR-490-3p*	[212]
TPT1-AS1	EOC tumor tissue samples (n=34) control tissue samples (n=20)	ES-2, SKOV3, HOSEpic	↑ expression in OC metastatic tissue and cell lines compared to controls	overexpression enhances proliferation, migration and invasion, *in vivo* facilitates intraperitoneal metastasis	TPT1, PI3K/AKT**	[213]
TTN-AS1	pairs of OC tumor tissue and control tissue (n=48)	SKOV3, A2780, OVCAR, HO8910, HOSEpiC, mouse xenograft	↑ expression in OC cell lines and tumor tissue compared to controls	knockdown inhibits proliferation, colony formation, invasion, migration, *in vivo*	miR-139-5p*	[214]

	TCGA-OV	A2780, OVCA429, IOSE80	↓ expression in OC tissue and cell lines	suppresses tumor formation overexpression inhibits proliferation, colony formation, promote apoptosis	miR-15b-5p*	[215]
TUG1	pairs of OC tumor and control tissue (n=62)	A2780, ES-2, OV-90, SKOV3, IOSE80	↑ expression in OC tissue compared to control tissue and cell lines	knockdown inhibits proliferation, colony formation, invasion, reversed EMT	E/N-cadherin, vimentin**	[216]
	pairs of OC tumor and control tissue (n=65)	IOSE80, A2780, SKOV3, ES-2, C3O	↑ expression in OC tissue, compared to control tissue and cell lines	knockdown inhibits proliferation, colony formation, migration, invasion, upregulation together with *MDM2*	MDM2**, miR-29b-3p*	[217]
UCA1	EOC tumor tissue samples (n=53) control tissue samples (n=29)	OMC685, A2780, SKOV3	↑ expression in OC tissue compared to control tissue and cell lines	knockdown reduced invasion, migration, downregulated *MMP14*	miR-485-5p*	[126]
UNC5B-AS1	TCGA-OV	IOSE-386, A2870, SW626, ES-2, SKOV3	↑ expression of OC tissue and cell lines	depletion of expression hinder proliferation, induce apoptosis	EZH2*	[218]
WDFY3-AS2	pairs of OC tumor and control tissue (n=30) datasets (GSE38666, GSE14407, GSE23383, GSE83693)	A2780, CP70, SKOV3, CAOV3, IOSE80, mouse xenograft	↓ expression in OC tissue compared to control	upregulation reduced tumor growth *in vivo*, suppresses proliferation, migration, invasion, EMT, enhanced apoptosis	miR-18a*	[219]
XIST	pairs of EOC tumor tissue and adjacent tissue (n=98)	OVCAR3, OV90, A2780, SKOV3, HOSE	↑ expression in OC tissue compared to control tissue and in OC cell lines compared to control cell lines	expression correlates with distant metastasis, stage and grade, *in vitro* silencing enhances proliferation, migration, invasion	-	[220]

* target (connection) predicted by bioinformatics tools like - TargetScan, DIANA-MicroT-CDS, miRWALK, miRDB, RNA22, PicTar, microRNA.org, PITA, miRNAnda, Starbase etc. and/or dual-luciferase assay. ** connection predicted by expression correlation

- not part of the study
